# Advancing Clinical Competence Through Interprofessional Education: Perspectives and Experiences of Healthcare Professionals and Clinical Educators

**DOI:** 10.3390/nursrep16070256

**Published:** 2026-07-21

**Authors:** Kholofelo Lorraine Matlhaba

**Affiliations:** Department of Health Studies, University of South Africa, Pretoria 0002, South Africa; matlhkl@unisa.ac.za; Tel.: +27-12-429-2073

**Keywords:** interprofessional education, clinical competence, healthcare professionals, nursing, public hospitals, South Africa, qualitative study, thematic analysis

## Abstract

**Background/Objectives:** Advancing clinical competence within contemporary healthcare systems requires innovative educational approaches that bridge professional silos. While Interprofessional Education (IPE) is globally recognized as a mechanism to enhance teamwork, its implementation within resource-constrained public health systems marked by historical and structural inequalities remains poorly understood. This study explored the perspectives and experiences of healthcare professionals and clinical educators regarding how interprofessional education (IPE) may contribute to clinical competence in selected South African public hospitals. **Methods:** A qualitative descriptive design was used. Semi-structured interviews were conducted with 22 purposively selected healthcare professionals and clinical educators from nursing, medicine, physiotherapy, occupational therapy, pharmacy, and social work. Interviews were conducted at selected hospitals following approval from hospital Chief Executive Officers. Data were collected by the researcher and co-researcher who is not part of this manuscript, audio-recorded, transcribed verbatim, and analyzed using thematic analysis. Ethical clearance and institutional permissions were obtained, and informed consent was secured from all participants. Trustworthiness was ensured through credibility, dependability, confirmability, and transferability. **Findings:** The thematic analysis yielded two primary patterns: (1) the perceived capacity of IPE to enhance clinical decision-making, communication, and collaborative practices; (2) systemic and structural constraints, including rigid professional hierarchies, severe time constraints, and a lack of formally institutionalized opportunities. **Conclusions:** IPE is perceived as an important contributor to collaborative clinical competence, but its sustainability depends on explicit institutional mandates. These findings imply that hospital managers and curriculum developers must transition from ad hoc interactions to structured, short-duration interdisciplinary platforms to overcome operational bottlenecks.

## 1. Introduction

Healthcare systems worldwide are becoming increasingly complex, requiring healthcare professionals who are able to work collaboratively to deliver safe, effective, and patient-centred care. In response to these evolving demands, interprofessional education (IPE) has been recognised as an important educational approach for preparing healthcare professionals for collaborative practice. IPE promotes learning that enables students and practitioners from different disciplines to learn with, from, and about one another in order to strengthen teamwork, communication, and clinical competence in practice. Effective collaboration is increasingly regarded as essential for improving patient outcomes, enhancing service delivery, and ensuring quality healthcare in complex clinical environments.

In South Africa, the need for collaborative practice is particularly important given the country’s historical legacy of apartheid, which resulted in persistent inequalities in healthcare access, workforce distribution, educational opportunities, and the availability of healthcare resources. Despite substantial health system reforms, many public healthcare facilities continue to experience shortages of healthcare professionals, limited infrastructure, high patient volumes, and inequitable access to healthcare services, particularly in underserved rural and peri-urban communities. These resource constraints place increasing demands on healthcare professionals to work effectively within multidisciplinary teams to optimize available resources and deliver comprehensive, patient-centred care. Consequently, IPE is increasingly recognized as a critical strategy for equipping future healthcare professionals with the collaborative competencies required to respond to these contextual challenges. However, despite growing recognition of the value of IPE, there is limited evidence regarding the perceptions and experiences of healthcare professionals and clinical educators on its role in enhancing clinical competence within South African clinical training settings, and understanding these perspectives is essential for informing the development and implementation of contextually relevant IPE initiatives that strengthen collaborative practice and improve healthcare delivery.

### 1.1. Background

Healthcare systems worldwide are becoming increasingly complex, requiring healthcare professionals who can work collaboratively to deliver safe, effective, and patient-centred care. As healthcare needs continue to evolve, effective teamwork, communication, and shared decision-making have become essential components of quality healthcare delivery [1]. In response to these demands, interprofessional education (IPE) has been widely recognized as an important educational approach for promoting collaboration, teamwork, communication, and clinical competence among healthcare professionals. IPE creates opportunities for healthcare professionals and students from different disciplines to learn with, from, and about one another, thereby fostering collaborative practice and improving healthcare outcomes [2]. Despite the recognized value of interprofessional education, its integration into healthcare education and clinical practice remains inconsistent across many settings. In numerous healthcare institutions, professionals continue to be trained within discipline-specific models that provide limited opportunities for shared learning and collaborative engagement. Consequently, challenges related to communication, teamwork, role clarification, and coordination of patient care may persist within healthcare environments. Organizational, structural, and professional barriers further constrain the effective implementation of interprofessional education and collaborative practice.

Several recent studies have demonstrated the positive contribution of interprofessional education in strengthening collaboration and clinical competence among healthcare professionals. In the recent study, ref. [3] found that interprofessional educational interventions contributed to sustained improvements in collaborative skills and motivation among nursing and medical students. Similarly, ref. [4] reported that participation in IPE programmes enhanced students’ perceptions of teamwork, communication, and interprofessional competencies. Furthermore, ref. [5] revealed that simulation-based IPE produced better collaborative learning outcomes compared to traditional single-profession educational approaches. In addition, ref. [6] highlighted the importance of interprofessional collaborative competencies in strengthening healthcare delivery within complex healthcare systems. It was further demonstrated that interprofessional learning experiences significantly improved communication skills, professional relationships, and collaborative attitudes among healthcare students [7].

Although the benefits of interprofessional education (IPE) have been increasingly documented in the literature, limited evidence exists regarding how healthcare professionals in South African public hospitals experience and perceive its role in enhancing collaboration, communication, teamwork, and clinical competence. Most existing studies have focused on educational interventions in academic settings or on single professional groups, leaving a gap in understanding the lived experiences of practicing healthcare professionals within the resource-constrained context of South African public healthcare.

This study addresses this gap by exploring the lived experiences of healthcare professionals from multiple disciplines regarding the role of interprofessional education in selected public hospitals in South Africa. By examining these experiences across diverse professional groups, the study provides context-specific insights into how IPE influences collaborative practice while identifying organizational factors that facilitate or hinder its implementation in routine clinical care. These findings contribute to the existing body of knowledge by informing the development of educational strategies, institutional policies, and collaborative healthcare practices that are responsive to the realities of South African public healthcare settings.

### 1.2. Aim of the Study

This study explored the perspectives and experiences of healthcare professionals and clinical educators regarding how interprofessional education (IPE) may contribute to clinical competence in selected South African public hospitals. The study examined how collaborative learning, teamwork, communication, and shared professional experiences contributed to the development of clinical knowledge, skills, attitudes, and patient-centred care among healthcare practitioners.

## 2. Materials and Methods

### 2.1. Research Design

This study adopted a qualitative research approach using a phenomenological design to explore the lived experiences of healthcare professionals regarding the role of IPE in enhancing clinical competence within selected public hospitals. A phenomenological design was considered appropriate because it seeks to understand how individuals experience and make meaning of a particular phenomenon within their natural contexts. This approach enabled an in-depth exploration of participants’ subjective experiences and the meanings they attributed to interprofessional education in their clinical practice [8,9]. The design enabled the researcher to obtain rich, contextualized accounts of how interprofessional education influences collaboration, communication, and clinical competence in practice.

The study was underpinned by the interpretivist paradigm, which assumes that reality is socially constructed and understood through individuals’ experiences and interpretations. This paradigm aligned with the phenomenological design by facilitating an in-depth understanding of participants’ lived experiences while recognizing that knowledge is co-constructed through interaction between the researcher and participants.

### 2.2. Study Setting and Participants

The study was conducted in selected public hospitals in South Africa. These hospitals were purposively selected because they provide multidisciplinary healthcare services and routinely employ healthcare professionals from various disciplines who collaborate in patient care. The hospitals comprise regional and tertiary-level institutions that provide comprehensive inpatient and outpatient services. Although interprofessional collaboration occurs daily, formal interprofessional education activities differ across hospitals, primarily offered by the Clinical Education and Training Units (CETUs). CETUs are departments within the hospitals mandated to coordinate, facilitate and provide quality assurance to all clinical learning activities. This organizational context provided an appropriate environment for exploring healthcare professionals’ lived experiences regarding the role of interprofessional education in enhancing clinical competence. Twenty-two healthcare professionals from multiple disciplines, including nursing, medicine, and allied health professions, participated in the study. The study did not aim to recruit equal numbers from each profession or hospital. Rather, participant recruitment was guided by the availability of eligible healthcare professionals who met the inclusion criteria and expressed willingness to participate. Consequently, the distribution of participants across professional groups and hospitals reflects practical accessibility rather than predetermined quotas. Recruitment continued until information power and thematic saturation were achieved, whereby no substantially new insights emerged from subsequent interviews. Participants were purposively selected based on their direct involvement in interprofessional collaborative practice within clinical settings. Purposive sampling was used to ensure the inclusion of information-rich cases relevant to the study objectives [10]. Inclusion criteria required participants to have at least one year of clinical experience and exposure to interprofessional teamwork in practice. Participation was voluntary, and only those who provided informed consent were included in the study. The study did not formally record the number of healthcare professionals who declined participation, as recruitment continued until thematic saturation was achieved.

### 2.3. Data Collection

Data were collected through semi-structured, in-depth interviews conducted by the researcher and co-researcher between May and June 2025. An interview guide was used to ensure consistency while allowing flexibility to explore emerging issues in greater depth. A pilot study was not conducted prior to data collection. The semi-structured interview guide was developed based on the study objectives and an extensive review of the relevant literature. Therefore, no interviews were conducted solely for pilot purposes; all interviews formed part of the final dataset and were included in the analysis. During data collection, the interviewer remained flexible and used probing questions where appropriate to elicit rich and comprehensive descriptions of participants’ perceptions and lived experiences. Interviews were conducted in private settings within the hospital environment to ensure confidentiality and minimize interruptions and lasted approximately 45 to 60 min each. Each interview was audio-recorded with participants’ consent and transcribed verbatim for analysis. Data collection continued until data saturation was achieved, defined as the point at which no new themes or insights emerged from successive interviews [11].

Data saturation was actively operationalized during the data collection process. The research team conducted concurrent thematic coding alongside ongoing interviews. Saturation was determined at the 19th interview, as no new codes or conceptual categories emerged regarding either the facilitators or systemic barriers of IPE; three additional interviews were conducted to confirm this thematic stability.

### 2.4. Data Analysis

Data were analyzed manually by both researchers, who are experienced qualitative researchers and followed the six-phase framework thematic analysis, which involved familiarization with the data, generating initial codes, searching for patterns, reviewing and refining themes, and defining and naming final themes [12]. Audio-recorded interviews were transcribed verbatim and checked against the recordings for accuracy before analysis. The analysis involved six iterative phases: (1) familiarization with the data through repeated reading of transcripts; (2) systematic generation of initial codes; (3) searching for potential themes by grouping conceptually related codes; (4) reviewing and refining themes against the coded extracts and the complete dataset; (5) defining and naming themes; and (6) producing the final report. This iterative process enabled a systematic and in-depth interpretation of participants’ experiences of interprofessional education. A primarily consensus-oriented coding approach was adopted. The two researchers independently coded an initial subset of transcripts before meeting to compare interpretations, discuss coding differences and develop a shared coding framework. The remaining transcripts were analyzed using this agreed coding framework while remaining open to the emergence of new codes where necessary. Throughout the analysis, reflexive discussions between the two researchers were undertaken to enhance consistency, reduce individual bias, and ensure that the emerging themes remained grounded in the participants’ narratives.

During data analysis, the researchers actively examined the dataset for disconfirming or divergent cases. Although participants described varying implementation challenges, no substantive divergent views were identified regarding the perceived value of interprofessional education.

The interviews were conducted by the first author, a female registered nurse with a doctoral degree in Health Sciences with Nursing Science and experience in qualitative research, together with a male co-researcher, a social worker holding a doctoral degree and experienced in qualitative research. Although the co-researcher is not an author of this manuscript, his contribution is acknowledged accordingly. Neither researcher had a prior professional relationship with the participants.

The researchers acknowledged that their professional backgrounds could potentially influence data collection and interpretation. To minimize this risk, they used a semi-structured interview guide, encouraged participants to elaborate on their responses, and employed probing questions throughout the interviews. Participants’ responses were regularly summarized and clarified during the interviews to ensure that their intended meanings were accurately understood.

The researchers functioned as external investigators (outsiders) with backgrounds in health professions education and public health. As they were not employed at the participating hospitals, the potential for institutional coercion or role-related influence was reduced. To enhance reflexivity, the researchers engaged in regular discussions throughout the data collection and analysis process to critically examine how their professional backgrounds, assumptions, and perspectives might influence data interpretation. These discussions helped ensure that the analysis remained grounded in participants’ accounts and that the final themes accurately reflected participants’ experiences rather than researchers’ preconceptions.

### 2.5. Measures of Trustworthiness

The trustworthiness of this qualitative study was ensured through the criteria of credibility, transferability, dependability, and confirmability [13]. Credibility was enhanced through prolonged engagement with participants and the use of in-depth, semi-structured interviews that allowed participants to freely express their lived experiences. Member checking was conducted by reading back summarized interpretations of the data to participants for verification, thereby ensuring that their perspectives were accurately represented.

Transferability was supported through the provision of thick, rich descriptions of the research context, participant characteristics, and study findings, enabling readers to determine the applicability of the findings to other similar healthcare settings.

Dependability was ensured through maintaining an audit trail of all research activities, including data collection procedures, interview guides, coding decisions, and thematic development. Peer debriefing with experienced qualitative researcher was also undertaken to review the consistency of the analysis process.

Confirmability was achieved through reflexive journaling by the researcher to minimize personal bias and ensure that the findings were grounded in the participants’ narratives rather than the researcher’s assumptions. In addition, all raw data, transcripts, and coding frameworks were securely stored to allow for verification and transparency of the analytic process.

### 2.6. Ethical Considerations

Ethical approval was obtained from the relevant institutional ethics committee prior to commencement of the study, and permission was granted by the hospital management of the selected study sites. Written informed consent was obtained from all participants prior to data collection. Participation was voluntary, and participants were informed of their right to withdraw at any stage without penalty. Confidentiality and anonymity were strictly maintained through the use of coded identifiers. All audio recordings, transcripts, and related documents were securely stored on password-protected devices accessible only to the research team. The study adhered to the ethical principles of respect for persons, beneficence, and justice throughout the research process. Care was taken to minimize any potential psychological discomfort during interviews, and participants were treated with dignity and respect throughout the research process. Findings were reported in a manner that ensured that no individual or institution could be identified.

## 3. Findings

### 3.1. Description of Study Participants

The study participants comprised healthcare professionals who were actively engaged in professional development and training within their respective specialties. The sample included doctors (2), nurses (4), social workers (2), pharmacists (4), dieticians (4), occupational therapists (2), speech therapists (2), physiotherapists (1), and one radiographer. The allied healthcare professional’s category consisted of six participants. All participants were drawn from four public hospitals located in a district within Gauteng Province. Among these hospitals, two were district hospitals, one was a tertiary hospital, and one was a regional hospital.

### 3.2. Presentation of Themes and Sub-Themes

This study identified two main themes and five subthemes. The themes and subthemes are outlined in Table 1 and are further described and discussed in the subsequent subsections.

#### 3.2.1. Theme 1: Transformative Role of Interprofessional Education in Advancing Clinical Competence

This theme describes participants’ perceptions of IPE as a transformative approach that strengthens clinical competence among healthcare professionals. Participants highlighted that learning and working collaboratively across professional disciplines contributed to improved knowledge sharing, critical thinking, and patient management. The findings suggest that IPE creates opportunities for healthcare professionals to learn from one another’s expertise, thereby enhancing the quality of care and fostering holistic patient-centred practices. Within this theme, participants particularly emphasized the role of IPE in improving decision-making as well as enhancing communication and collaboration in clinical settings.

These findings suggest that participants viewed clinical competence as a collective outcome of shared learning rather than an individual professional achievement. The experiences described indicate that collaborative learning strengthened participants’ confidence in integrating diverse professional perspectives to improve patient care. Although these benefits were recognized across all professional groups, nurses frequently emphasized improved coordination of patient care, whereas allied health professionals highlighted increased recognition of their roles within multidisciplinary teams.

##### Sub-Theme 1.1: Improved Decision-Making

Participants described interprofessional education as an important mechanism for improving clinical decision-making processes. Through shared learning experiences and collaborative practice, healthcare professionals were able to draw on diverse professional perspectives when managing patient care. Participants indicated that exposure to multiple viewpoints enhanced problem-solving abilities, reduced uncertainty in clinical judgments, and promoted more informed and comprehensive decisions. The findings further revealed that collaborative decision-making contributed to improved patient outcomes and strengthened confidence among healthcare professionals in managing complex clinical situations.

The structural friction between professional groups manifests clearly in prescribing patterns. For instance, a pharmacist highlighted how targeted, brief educational interventions actively reshape clinical practices:

*“If you understand what other professionals do, you get a holistic view of the patient care whether medical, social, psychological and can provide more effective interventions.”*

A social worker said, *“Even as a social worker, I try to understand the patient’s medical condition by researching diagnoses so I can align my support with their clinical needs.”*

Another one said, *“Understanding the roles of others gives a holistic view of the patient care, medical, psychological, social and helps guide clinical decisions.”*

##### Sub-Theme 1.2: Enhanced Communication and Collaboration

Effective communication and collaboration emerged as central outcomes of interprofessional education. Participants reported that IPE encouraged open communication among healthcare professionals and promoted mutual understanding of each profession’s roles and responsibilities. The findings demonstrated that collaborative interactions enhanced teamwork, reduced misunderstandings, and improved coordination of patient care activities. Participants further highlighted that stronger communication channels fostered professional respect, trust, and shared accountability, which ultimately supported the delivery of integrated and patient-centred healthcare services.

A physiotherapist said, *“I prefer to document in the patient’s file and call the referring professional to explain why a referral might not be appropriate that kind of clear communication prevents misunderstandings.”*

Another physiotherapist said, *“I join the doctors’ meetings in the mornings so I can raise any challenges and clarify who needs to do what.”*

A pharmacist said *“With the doctors we look at rational prescribing, if we notice trends or contraindications, we have a training with the doctors and re-inform them. “I feel it was very fruitful training and prescribing patterns changed after that.”*

Another pharmacist said, *“We have created a platform for weekly interaction through Friday meetings with doctors. Instead of long, formal training, we do shorter, focused discussions for 15 or 20 min, just touching base. That has been very beneficial.”*

#### 3.2.2. Theme 2: Systemic and Structural Constraints

This theme captures the systemic and structural barriers that limited the effective implementation and sustainability of interprofessional education within healthcare settings. Although participants recognized the value of IPE in strengthening clinical competence, they also identified organizational and contextual challenges that hindered consistent collaboration and learning opportunities. The findings revealed that institutional limitations, professional hierarchies, and workload pressures negatively influenced the integration of interprofessional practices in everyday clinical environments. Sub-themes associated with this theme included limited structured opportunities, hierarchical dynamics, and time and organizational constraints.

The findings further suggest that these barriers were interrelated, with organizational limitations reinforcing professional hierarchies and reducing opportunities for meaningful interprofessional engagement. Participants’ experiences indicate that the successful implementation of IPE depends not only on individual willingness to collaborate but also on institutional commitment to creating supportive learning environments. While these challenges were experienced across all professional groups, nurses more frequently highlighted workload pressures, whereas allied health professionals emphasized limited opportunities to participate fully in multidisciplinary collaboration.

##### Sub-Theme 2.1: Limited Structured Opportunities

Participants identified the lack of formally structured opportunities for interprofessional engagement as a major challenge. Although collaborative learning occasionally occurred in practice, participants indicated that such interactions were often informal, inconsistent, and dependent on individual initiative rather than institutional support. The findings revealed that the absence of regular interdisciplinary training sessions, workshops, and collaborative clinical activities limited opportunities for sustained interprofessional learning and professional development. Participants expressed the need for more intentional and structured approaches to support continuous interprofessional engagement within healthcare institutions.

A Nurse said: *“You want all the staff to have the info. But you cannot have the same training three times.”*

Another Nurse said: *“You are told to train your staff; you have got an hour to train on a workshop that was meant for three days.”*

A dietician said: *“Doctors do not always understand what is the role that the dietician, for example, their role in planning care of patient.”*

##### Sub-Theme 2.2: Hierarchical Dynamics

Hierarchical relationships within healthcare settings were reported as barriers to effective interprofessional collaboration. Participants described how professional hierarchies sometimes restricted open communication and limited equal participation among members of the healthcare team. In some instances, certain professional groups were perceived to dominate decision-making processes, which negatively affected teamwork and mutual respect. The findings suggest that hierarchical dynamics may undermine collaborative practice by creating power imbalances that discourage active participation and knowledge sharing among healthcare professionals.

One dietician said: *“So if you have that support also from higher up It will also give you more confidence to be open and not feel that you are going to be condemned with scolded at and it also helps you to grow as an individual.”*

Another dietician said: *“I was saying that it is important for us to know each other’s roles and what you do. So, I need to know as a professional, what is a social worker’s role, what is a psychologist, but what is the difference between the two. So, when I have an issue that I do not send the patient to social worker while the patient needed psychology.”*

A pharmacist said: *“We also have meetings only with doctors, if we included allied departments, it could improve a lot.”*

Another pharmacist said: *“We had one other training with the doctors in between, but it is not always possible. But the meeting provides a platform for communication regarding prescribing or rational medicine use. It kind of opened the eyes to certain things. It is about communication; it is about understanding things from different perspectives.”*

##### Sub-Theme 2.3: Time and Organizational Constraints

Time limitations and organizational pressures were frequently highlighted as obstacles to interprofessional education and collaboration. Participants reported that heavy workloads, staff shortages, and demanding clinical responsibilities reduced opportunities for healthcare professionals to engage in collaborative learning activities. Additionally, organizational factors such as inadequate scheduling, limited managerial support, and insufficient resources further constrained the implementation of interprofessional initiatives. These findings indicate that operational challenges within healthcare settings can significantly affect the integration and sustainability of interprofessional education practices.

One pharmacist said: *“So if there could be a weekly interaction, maybe instead of a formal training that could take an hour, you could maybe make more frequent but shorter. And just an interaction, just touching base. And if we maybe can be a different topic, whatever or you will discuss whatever challenges you faced in that week”.*

Another pharmacist said: *“There’s no way we can shut down pharmacy and all the pharmacy staff can be trained at once.”*

A Social worker said: *“Maybe instead of a formal training that could take an hour, you could maybe make more frequent but shorter interactions.”*

A physiotherapist said: *“It is a small facility and only two are employed, you are on your own. More staff can improve and make things very effective.”*

A medical doctor said: *“Some people are quite rushed… but things happen. There is emergency space. So I think that is maybe the biggest obstacle.”*

## 4. Discussion

The findings of this study highlighted the significant contribution of IPE in advancing clinical competence among healthcare professionals and clinical educators. Participants described IPE as a transformative educational and collaborative approach that strengthened decision-making, enhanced communication, and promoted collaborative practice within healthcare settings. However, the findings also revealed persistent systemic and structural barriers that limited the effective implementation of IPE, including inadequate structured opportunities, hierarchical professional dynamics, and organizational and time-related constraints. These findings align with existing literature emphasizing that while IPE has the potential to improve healthcare quality and professional competence, its successful implementation remains dependent on institutional support, organizational culture, and collaborative healthcare systems.

### 4.1. Transformative Role of Interprofessional Education in Advancing Clinical Competence

The findings demonstrated that IPE played a transformative role in strengthening clinical competence by improving healthcare professionals’ decision-making abilities. Participants reported that collaborative learning environments enabled practitioners from different disciplines to share knowledge, discuss patient management strategies, and collectively solve complex clinical problems. Through interprofessional interactions, healthcare professionals developed broader perspectives regarding patient care, which enhanced critical thinking and informed clinical judgement. These findings are consistent with previous studies indicating that IPE fosters collaborative decision-making and improves healthcare professionals’ ability to manage complex patient needs effectively [14,15,16].

The improvement in clinical decision-making identified in this study may be attributed to the integration of diverse professional expertise during patient management. Participants explained that exposure to different professional viewpoints enabled them to better understand the interconnected nature of healthcare delivery and the importance of coordinated care. Similar findings have been mentioned in some official reports [17,18], which emphasized that collaborative learning enhances practitioners’ confidence, accountability, and clinical reasoning. In multidisciplinary healthcare environments, patient outcomes often depend on the collective competence of healthcare teams rather than individual professional expertise alone. Therefore, IPE contributes to safer, evidence-based, and patient-centred decision-making processes. The findings regarding improved decision-making align with Étienne Wenger’s framework of Communities of Practice. When healthcare professionals interact across disciplinary lines, they negotiate a ‘shared repertoire’ of clinical knowledge. This collective learning breaks down professional isolation and expands individual clinical reasoning.

The findings further revealed that IPE enhanced communication and collaboration among healthcare professionals. Participants described how interprofessional interactions improved mutual understanding of professional roles, strengthened teamwork, and facilitated open communication in clinical settings. Effective communication is widely recognized as a fundamental component of safe and quality healthcare delivery. The findings support existing evidence suggesting that communication failures among healthcare professionals contribute significantly to medical errors, fragmented care, and poor patient outcomes [19]. Through IPE, participants reported gaining confidence in communicating with colleagues from other disciplines, which improved coordination of care and reduced misunderstandings in clinical practice.

The enhancement of collaboration observed in this study aligns with the principles of collaborative practice proposed by the World Health Organization (WHO), which emphasize that healthcare professionals who learn together are more likely to work effectively together [20]. This is further supported by a systematic review study [21], which indicated that IPE created opportunities for knowledge exchange, respect for professional diversity, and shared responsibility in patient care. Such collaborative relationships are essential in modern healthcare systems characterized by increasingly complex patient conditions requiring multidisciplinary interventions. Similar findings have been reported, indicating that IPE enhances teamwork competencies, promotes role clarification, and strengthens interprofessional relationships, ultimately improving healthcare service delivery [22,23,24].

The findings also demonstrated that IPE contributed to the development of patient-centred care approaches. Participants reported that collaborative practice enabled healthcare professionals to address patient needs more holistically by integrating perspectives from multiple disciplines. Patient-centred care requires healthcare providers to work collaboratively to ensure continuity, safety, and comprehensive management of patient conditions. This finding corroborates studies indicating that IPE promotes holistic care and enhances patient satisfaction through coordinated healthcare delivery [25,26]. Participants recognized that collaborative practice reduced duplication of services and improved the efficiency of patient management processes.

Furthermore, participants described IPE as an important mechanism for professional growth and lifelong learning. Through interprofessional interactions, healthcare professionals gained opportunities to learn from one another’s expertise, experiences, and clinical approaches. This finding reflects social learning theory, which posits that learning occurs through interaction, observation, and shared experiences within social contexts [27]. In healthcare settings, collaborative learning environments facilitate continuous professional development and competency enhancement. The findings support previous research suggesting that IPE promotes reflective practice, professional confidence, and adaptive learning among healthcare practitioners [2,28].

The positive perceptions of IPE identified in this study also suggest that collaborative education may contribute to reducing professional isolation and promoting supportive workplace cultures. Participants described how interprofessional engagement fostered mutual respect, trust, and professional recognition among team members. This finding is particularly relevant in healthcare systems where professional silos often hinder effective collaboration. It is suggested that IPE has the potential to transform organizational cultures by encouraging cooperative relationships and reducing professional stereotypes [29,30]. Consequently, IPE may serve as a strategic approach for strengthening teamwork and enhancing workforce cohesion within healthcare institutions.

### 4.2. Systemic and Structural Constraints

Despite the positive contributions of IPE, participants identified several systemic and structural barriers that limited its implementation and effectiveness. One of the major challenges highlighted in this study was the limited availability of structured opportunities for interprofessional learning and collaboration. Participants reported that IPE activities were often informal, inconsistent, or dependent on individual initiative rather than institutional planning. The lack of formalized IPE programmes limited opportunities for healthcare professionals and students to engage in collaborative learning experiences. Similar findings have been reported globally, where inadequate curriculum integration and insufficient institutional support remain significant barriers to IPE implementation [20,22].

The absence of structured IPE opportunities may negatively affect the sustainability and effectiveness of collaborative practice initiatives. Participants emphasized the need for organised training sessions, multidisciplinary meetings, simulation activities, and collaborative clinical teaching strategies to strengthen interprofessional learning. Existing literature suggests that structured IPE interventions are more effective in promoting teamwork competencies and improving patient outcomes than informal collaboration alone [31,32]. Therefore, healthcare institutions and educational programmes should prioritize the integration of structured IPE frameworks within professional training and clinical practice environments.

Another significant finding of this study was the influence of hierarchical professional dynamics on interprofessional collaboration. Participants reported that traditional professional hierarchies often created power imbalances that limited open communication and equal participation within healthcare teams. In some instances, certain professional groups were perceived as having greater authority in decision-making processes, which undermined collaborative practice. These findings are consistent with previous studies demonstrating that hierarchical cultures within healthcare systems can negatively affect teamwork, communication, and mutual respect among professionals [33,34,35].

Hierarchical dynamics may hinder the development of effective interprofessional relationships by reinforcing professional boundaries and limiting shared decision-making. Participants described situations where junior professionals or members of less dominant disciplines felt excluded from clinical discussions or hesitant to contribute their perspectives. Such experiences may reduce confidence, discourage collaboration, and compromise patient-centred care. Healthcare hierarchies often perpetuate communication barriers and inhibit collaborative learning [36]. Addressing these hierarchical structures requires organizational commitment to fostering inclusive workplace cultures that value the contributions of all healthcare professionals equally.

The findings also highlighted time and organizational constraints as major barriers to effective IPE implementation. Participants explained that heavy workloads, staff shortages, limited resources, and demanding clinical schedules reduced opportunities for collaborative learning and teamwork. In busy healthcare environments, professionals often prioritized immediate patient care responsibilities over interprofessional engagement activities. Similar barriers have been widely documented in the literature, particularly in resource-constrained healthcare settings where operational pressures limit opportunities for professional collaboration [37].

Time constraints may significantly affect the sustainability of IPE initiatives because collaborative learning requires dedicated planning, coordination, and participation. Participants suggested that organizational support is essential for creating protected time for interprofessional meetings, training, and reflective practice sessions. Previous studies emphasized that successful IPE implementation requires leadership support, adequate resources, and institutional policies that promote collaborative practice [38,39]. Without such support, healthcare professionals may struggle to engage consistently in interprofessional learning activities.

Organizational culture significantly influences the implementation of IPE. Limited leadership support, unclear policies, and weak institutional commitment hinder collaborative practice and teamwork, highlighting organizational readiness and leadership engagement as key determinants of successful integration [40]. Findings show that embedding collaborative learning through simulation, multidisciplinary engagement, and clinical placements enhances communication, teamwork, and shared decision-making among healthcare students and practitioners. However, despite these benefits, persistent organizational and systemic barriers continue to limit consistent implementation, affecting the sustainability of IPE and its contribution to clinical competence and patient-centred care across academic and clinical settings.

### 4.3. Implications for Advanced Practice Nursing

The findings of this study have important implications for Advanced Practice Nursing (APN), particularly in strengthening interprofessional collaboration, clinical leadership, and competency-based practice. According to the International Council of Nurses (ICN), APNs are expected to demonstrate advanced clinical expertise while leading evidence-based practice, promoting interprofessional collaboration, mentoring healthcare professionals, and contributing to quality improvement initiatives [41]. Advance practice nursing models and frameworks identify collaboration, leadership, evidence-based practice, education, and advanced clinical decision-making as core competencies required of Advanced Practice Nurses [42,43,44,45]. From this study, the perceived improvements in communication, teamwork, shared decision-making, and clinical confidence reported by participants suggest that interprofessional education may foster these competencies, supporting preparation for advanced nursing practice. Furthermore, APNs are strategically positioned to champion interprofessional learning, facilitate collaborative practice, mentor less experienced clinicians, and lead educational initiatives that strengthen patient-centred care and health system performance. These findings are consistent with evidence identifying clinical leadership and collaborative practice as fundamental competencies of APNs and support the integration of IPE into continuing professional development to prepare nurses for advanced practice roles. Nursing leaders must be prepared with foundational transformational leadership competencies in order to meet the challenges of leading collaboratively with other professions.

### 4.4. Recommendations

#### 4.4.1. Recommendations for Higher Education Institutions

Higher education institutions should strengthen the integration of interprofessional education in undergraduate and postgraduate healthcare curricula. Interdisciplinary activities such as simulations, case-based learning, collaborative clinical placements, and team-based projects should be used to develop communication, teamwork, and shared decision-making skills. Institutions should also provide ongoing professional development for educators and establish partnerships with healthcare facilities to ensure consistent integration of interprofessional education in both academic and clinical settings.

#### 4.4.2. Recommendations for Healthcare Facilities

Healthcare facilities should promote supportive environments that encourage interprofessional collaboration and continuous learning. Management should implement structured training, multidisciplinary ward rounds, and collaborative case discussions to strengthen teamwork and clinical competence. Institutions should also address hierarchical barriers by fostering mutual respect, inclusivity, and shared accountability. Adequate resources, staffing, and time should be allocated to support interprofessional education, while policies and frameworks promoting collaborative practice should be developed and regularly monitored for sustainability and effectiveness.

#### 4.4.3. Recommendations for Future Research

Future research should examine the long-term effects of interprofessional education on patient outcomes, healthcare delivery, and professional practice across diverse settings. Quantitative and mixed-methods studies are needed to evaluate its impact on clinical competence, teamwork, and communication. Broader studies with larger and more diverse samples would improve generalizability, while further research should explore ways to address organizational and hierarchical barriers to implementation. Including the perspectives of students, patients, and healthcare managers could also provide a more comprehensive understanding of interprofessional education in healthcare systems.

### 4.5. Study Limitations

This study possesses several limitations that qualify the scope of its findings. First, the sample size of 22 participants across nine distinct professions means that certain disciplines (such as physiotherapy and radiography) were represented by only a single participant, preventing robust cross-professional comparisons. Second, the study relied solely on self-reported interview data, which introduces potential social desirability bias, as participants may have overreported their alignment with collaborative practices. The study lacks direct clinical observation or methodological triangulation to verify whether these perceived competencies manifest in actual behavioral changes. Finally, the qualitative design was bounded within public hospitals in a single district of Gauteng Province, limiting the direct generalizability of the findings to private or rural healthcare contexts.

### 4.6. Study Strengths

This study’s strength lies in its phenomenological approach, which provided deep insights into healthcare professionals’ experiences of interprofessional education. The inclusion of diverse professional disciplines enriched understanding of collaboration, communication, and clinical competence in healthcare settings. The study also adds valuable knowledge to the limited South African literature on interprofessional education in public hospitals and offers practical guidance for curriculum development, institutional policies, and improved patient care practices.

## 5. Conclusions

Interprofessional education plays a vital role in improving clinical competence by enhancing collaboration, communication, teamwork, and role clarification among healthcare professionals. The study found that interprofessional learning strengthens clinical decision-making, supports patient-centred care, and improves professional relationships within healthcare settings. Although challenges to implementation exist, effective institutional support can enhance its success and contribute to comprehensive, high-quality healthcare delivery.

## Figures and Tables

**Table 1:** Themes and sub-themes generated from data analysis.

Themes	Sub-Themes
1. Transformative role of Interprofessional Education in advancing clinical competence	Improved decision-making Enhancing communication and collaboration
2. Systemic and structural constraints	Limited structured opportunities Hierarchical dynamics Time and organizational constraints

## Data Availability

Data are available from the corresponding author upon reasonable request due to privacy and ethical reasons.

## Public Involvement Statement

There was no public involvement in any aspect of this research.

## Guidelines and Standards Statement

This study was reported in accordance with the Consolidated Criteria for Reporting Qualitative Research (COREQ): A 32-item Checklist for Interviews and Focus Groups [46]. This was to ensure comprehensive and transparent reporting of the design, data collection, analysis, and finding.

## Abbreviations

The following abbreviations are used in this manuscript:

IPE	Interprofessional Education
CREC	College Research Ethics Committee
COREQ	Criteria for Reporting Qualitative Research
WHO	World Health Organization
